# Radiolabeled PSMA Inhibitors

**DOI:** 10.3390/cancers13246255

**Published:** 2021-12-13

**Authors:** Oliver C. Neels, Klaus Kopka, Christos Liolios, Ali Afshar-Oromieh

**Affiliations:** 1 Institute of Radiopharmaceutical Cancer Research, Helmholtz-Zentrum Dresden-Rossendorf (HZDR), Bautzner Landstrasse 400, 01328 Dresden, Germany; k.kopka@hzdr.de; 2 Faculty of Chemistry and Food Chemistry, School of Science, Technical University Dresden, Mommsenstrasse 4, 01062 Dresden, Germany; 3 Department of Pharmaceutical Chemistry, Faculty of Pharmacy, National & Kapodistrian University of Athens, Zografou, 15771 Athens, Greece; liolios.xr@gmail.com; 4 INRASTES, Radiochemistry Laboratory, NCSR “Demokritos”, Ag. Paraskevi Attikis, 15310 Athens, Greece; 5 Department of Nuclear Medicine, Bern University Hospital (Inselspital), Freiburgstrasse 18, 3010 Bern, Switzerland; ali.afshar@insel.ch

**Keywords:** PSMA, prostate-specific membrane antigen, PSMA inhibitor, radiolabeling, PET, SPECT, fluorescence, endoradiotherapy, theranostics, radioguided surgery, fluorescence-guided surgery, targeted photodynamic therapy, radionuclides

## Abstract

**Simple Summary:**

Prostate cancer remains one of the leading causes of cancer death in men worldwide. Despite the recent success in the development and clinical application of radiopharmaceuticals targeting the prostate-specific membrane antigen (PSMA) for diagnosis and endoradiotherapy of prostate cancer, more research is ongoing to further investigate and improve patient care and quality of life. Herein, an overview of novel developments and applications for small molecule and low-molecular weight radiolabeled PSMA inhibitors with an outlook to clinical translation is given.

**Abstract:**

PSMA has shown to be a promising target for diagnosis and therapy (theranostics) of prostate cancer. We have reviewed developments in the field of radio- and fluorescence-guided surgery and targeted photodynamic therapy as well as multitargeting PSMA inhibitors also addressing albumin, GRPr and integrin α_v_β_3_. An overview of the regulatory status of PSMA-targeting radiopharmaceuticals in the USA and Europe is also provided. Technical and quality aspects of PSMA-targeting radiopharmaceuticals are described and new emerging radiolabeling strategies are discussed. Furthermore, insights are given into the production, application and potential of alternatives beyond the commonly used radionuclides for radiolabeling PSMA inhibitors. An additional refinement of radiopharmaceuticals is required in order to further improve dose-limiting factors, such as nephrotoxicity and salivary gland uptake during endoradiotherapy. The improvement of patient treatment achieved by the advantageous combination of radionuclide therapy with alternative therapies is also a special focus of this review.

## 1. Introduction

Radiopharmaceuticals targeting the prostate-specific membrane antigen (PSMA) have seen great success in Nuclear Medicine for both diagnosis and endoradiotherapy of prostate cancer (PCa) [1,2] thus paving the way for (radio)theranostics [3,4,5] with guidelines being published by international experts and societies [6,7,8,9,10,11,12,13,14,15,16,17,18,19].

[^68^Ga]Ga-PSMA-11 (gozetotide) [20,21,22,23] and [^18^F]DCFPyL (piflufolastat) [24,25,26,27] as diagnostic imaging agents have been approved in the US by the Food and Drug Administration (FDA) since 1st December 2020 [28,29,30,31,32] and 27th May 2021 [33,34], respectively. Meanwhile, [^18^F]PSMA-1007 [35,36,37,38] is awaiting regulatory approval in European countries pending a phase 3 clinical study being completed (NCT04102553). [^99m^Tc]Tc-MIP-1404 (trofolostat) is a highly promising SPECT agent [39,40,41,42]. Monographs have been published by the European Directorate for the Quality of Medicines (EDQM) for [^68^Ga]Ga-PSMA-11 (Ph. Eur. Monograph 3044) and [^18^F]PSMA-1007 (Ph. Eur. Monograph 3116) which came into force 1st April 2021 and 1st July 2021, respectively [43]. Alberts et al. recently reported an overview that evaluated the most commonly used PET radiopharmaceuticals for the diagnosis of prostate cancer [44].

The most prominent ligands used for PSMA-targeted endoradiotherapy with beta^−^ and alpha emitters are PSMA I&T [45,46,47,48] and PSMA-617 (vipivotide tetraxetan) [49,50,51,52]. The latter radiopharmaceutical is expected to receive marketing authorization in several countries after successfully accomplishing a phase 3 clinical study using [^177^Lu]Lu-PSMA-617 (NCT03511664) [53,54,55]. In response to this predicted breakthrough, infrastructural challenges should be addressed in the capacity planning of Nuclear Medicine therapy departments in the future [56]. Moreover, the experience of patients enrolled in a clinical trial involving radioactive treatment [57] and the necessity of a proper patient selection by PET imaging prior endoradiotherapy demands further attention [58,59,60].

In the future, a much broader acceptance and application of PSMA theranostics can be anticipated when diagnosis and treatment are being reimbursed by health insurances [61]. Amongst the aforementioned radiopharmaceuticals, several other radioligands targeting PSMA are either under clinical investigation or have been translated into a clinical setting (Table 1) [19,62]. The combination of radionuclide therapy with other treatment strategies offers new opportunities for patient care, these potential alternatives have been reviewed by Sandhu et al. and Zhang et al. [63,64]. This review aims to provide an overview of novel developments and applications in the area of small-molecule and low-molecular weight-based radiolabeled PSMA inhibitors [65], keeping their prospective clinical translation in mind.

## 2. Quality and Technical Improvements of PSMA Inhibitors

### 2.1. Quality Issues and Technical Improvements

Although radiolabeled PSMA inhibitors are used extensively for clinical applications, including radiotracers for which quality standards have been established by legally applicable specific monographs [43], special care needs to be taken during radiosynthesis and quality control of the radiopharmaceuticals.

Iudicello et al. showed that PSMA-11, which is used as a reference standard and precursor for the synthesis of [^68^Ga]Ga-PSMA-11, when dissolved in acidic aqueous solution forms a ^nat^Fe-PSMA-11 complex (a side product with two diastereomeric forms similar to the ones previously reported for [^68^Ga]Ga-PSMA-11) resulting thus, in lower radiochemical yield, chemical purity and radiochemical purity [91,92]. As a consequence, for storage of the PSMA-11 solution, EDTA was added to transchelate the Fe iron from the HBED-CC chelator.

Five-membered ring systems can be formed as radioactive side-products by a thermally mediated and pH-dependent condensation reaction of the Glu-urea-Lys binding motif during synthesis of [^177^Lu]Lu-PSMA-617 [93]. These side-products did not show any binding affinity to PSMA and were rapidly excreted via the kidneys from the body of five patients. The synthesis of [^177^Lu]Lu-PSMA-617 could be optimized by adjustment of pH and temperature and subsequent reduction of side-products, however, the risk of formation of unwanted radioactive side-products for PSMA inhibitors bearing the Glu-urea-Lys binding motif still remains [93].

Reaction temperature during radiolabeling plays an important role as shown above. Attempts have been made to replace DOTA, which requires time and heating for complexing radiometals, with a different chelator that allows low temperature radiolabeling in particular for the alpha-particle emitting isotope ^225^Ac. Thiele et al. showed that the eighteen-membered macrocyclic chelator macropa is suitable to complex ^225^Ac at room temperature (RT) in 5 min with high stability in human serum over 8 days [94]. Macropa was then conjugated to the PSMA/albumin binding ligand RPS-070 and radiolabeled with ^225^Ac within 20 min at RT. [^225^Ac]Ac-macropa-RPS-070 was tested in LNCaP tumor bearing mice where it showed high in vivo stability and high kidney uptake after 4 h (52 ± 16% ID/g), which then cleared rapidly, while tumor uptake (13 ± 3% ID/g) washout was slower. Reissig et al. expanded the “macropa”-principle by adding one or two alkyne moieties for conjugation of biomolecules using the copper-catalyzed azide-alkyne-cycloaddition [95]. A PSMA binding block was attached via click chemistry to obtain either the monomer mcp-M-PSMA or the dimer mcp-D-PSMA. Radiolabeling was done within 1 h at RT, both [^225^Ac]Ac-mcp-M-PSMA and [^225^Ac]Ac-mcp-D-PSMA were evaluated in vitro and in vivo using LNCaP cells and the corresponding xenograft mouse model. It is noticeable that the tumor uptake for the monomer was higher within the first hour and then decreased while the tumor uptake for the dimer constantly increased within 24 h making it more suitable for a therapy approach [95].

Radiopharmaceuticals are also prepared via so-called cold kits, for a long time established in Nuclear Medicine by reconstitution with the eluate of ^99m^Tc generators [96]. More recently, this approach has seen a wider application also for PET radiopharmaceuticals with three commercially available kits using either PSMA-11 (Illuccix and isoPROtrace-11) or THP-PSMA (GalliProst) for radiolabeling with the eluate of a ^68^Ge/^68^Ga generator [97]. Illuccix has been approved by the Australian Therapeutic Goods Administration (TGA) on 2nd November 2021 [98].

### 2.2. Salivary Gland Uptake

Uptake in the salivary glands is the dose-limiting factor during endoradiotherapy with small-molecule PSMA inhibitors, resulting in (partially reversible) xerostomia when applied with ^177^Lu, and severe to persistent xerostomia when applied with ^225^Ac [50,51]. The quality of life for patients can, therefore, be significantly impaired despite preventive strategies, e.g., injection of botulinum toxin, external cooling, or gustatory stimulation have been described [99,100,101,102,103]. The uptake mechanism of PSMA inhibitors in salivary glands has not been thoroughly investigated to date, a more comprehensive review concerning known mechanisms is described by Heynickx et al. [103]. Tönnesmann et al. showed in quantitative autoradiography experiments that in the salivary glands of pigs for small-molecule and low-molecular weight PSMA inhibitors the uptake is highly non-specific and at the same time specific while the exact ratio is not yet understood [104]. Attempts to minimize the uptake of the PSMA-targeted radiopharmaceutical in the salivary glands have been made following the modifications at the inhibitor component. This resulted in low salivary gland uptake and very low tumor uptake [105]. Two independent preclinical studies demonstrated that additional substance amount-dependent administration of nonradioactive PSMA ligand (PSMA-11 or DCFPyL, respectively) and reduction of the effective molar activity significantly reduces the radioligand uptake ([^177^Lu]Lu-PSMA-617 or [^18^F]DCFPyL, respectively) in the salivary glands (Table 2) [106,107]. However, these promising results need to be confirmed in further preclinical and clinical studies.

## 3. PSMA Inhibitors for Guided Surgery

### 3.1. PSMA Inhibitors for Radioguided Surgery

In addition to the properties for noninvasive single photon emission computed tomography (SPECT) or positron emission tomography (PET) imaging, the radiation exposed from the radionuclide bound to the PSMA inhibitor can also be used for the detection of tumor entities during intraoperative surgery using a gamma probe in a practice named radioguided surgery (RGS) [108].

PSMA I&T and PSMA-617 radiolabeled with ^111^In were initially used for SPECT imaging and RGS [75,79,80,109,110], with both showing detection rates lower than [^68^Ga]Ga-PSMA-11 [111]. However, due to the limited availability of [^111^In]InCl_3_, PSMA I&T derived Mas_3_-y-nal-k(Sub-KuE) (PSMA I&S) was developed and radiolabeled with ^99m^Tc [81]. [^99m^Tc]Tc-PSMA I&S RGS using a gamma probe was applied in a clinical setting after identifying tumor lesions with [^68^Ga]Ga-PSMA-11 PET [112,113]. In a study including 210 PCa patients, high detection rates were found for [^99m^Tc]Tc-PSMA I&S for PSA levels > 4 ng/mL [114]. The radiation dosimetry of [^99m^Tc]Tc-PSMA I&S, which was performed in a single-center study, resulted in comparable effective doses with other ^99m^Tc radiopharmaceuticals and lower effective doses than either ^68^Ga- or ^18^F-radiolabeled PSMA inhibitors ([^99m^Tc]Tc-PSMA I&S: 0.0052 mSv/MBq, [^68^Ga]Ga-PSMA-11: 0.0236 mSv/MBq, [^18^F]PSMA-1007: 0.022 mSv/MBq) [115,116,117].

As an undesirable consequence of RGS, personnel are typically exposed to constant radiation from the radiopharmaceutical. The occupational radiation exposure for workers involved in RGS using [^99m^Tc]Tc-PSMA I&S was determined with a maximum dose of 0.32 mSv/year for 100 procedures [118]. When laparoscopic gamma probes are used for sentinel lymph node dissection [119] they suffer from practical limitations, due to rotational impairment of the probe [120]. This limitation has been circumvented by the development of a drop-in probe, which can be controlled by grasps of the DaVinci robotic-assisted surgery system, thereby providing additional rotational freedom and reducing the radiation dose [121]. A first-in-human application has been reported for [^99m^Tc]Tc-PSMA I&S after preoperative [^68^Ga]Ga-PSMA-11 PET/CT [122]. In an experimental study with seven patients, the drop-in concept was tested with [^68^Ga]Ga-PSMA-11 using a beta probe instead of a gamma probe. After an additional dose of approximately 70 MBq [^68^Ga]Ga-PSMA-11 per patient prior surgery, the obtained surgical specimens were evaluated ex vivo using a DaVinci robotic system equipped with the drop-in beta probe [123]. Radiation dose to workers limits the use to 62 procedures/year of this technique, but improvements towards a lower occupational radiation dose are feasible. Alternative imaging technologies focusing on beta-radiation emission, e.g., Cerenkov luminescence imaging using [^68^Ga]Ga-PSMA-11 are currently under early clinical investigation [124,125,126,127,128,129,130].

### 3.2. PSMA Inhibitors for Fluorescence-Guided Surgery and/or Targeted Photodynamic Therapy

Aside from radionuclide detection for radioguided surgery, an attractive alternative for intraoperative guided surgery is radiolabeled molecules functionalized with fluorescent dyes for optical/fluorescence imaging [131,132,133,134]. A variety of commercially available dyes with different absorptions and emission spectra used for dual-labeled imaging agents has been reported [135]. Dual-labeled agents allow preoperative nuclear imaging (prestaging/staging) and subsequent treatment planning while fluorescence imaging makes surgical tumor resection possible and more accurate due to the high spatial resolution.

Baranski et al. studied the PSMA-11 derived hybrid compound Glu-urea-Lys-HBED-CC-IRDye800CW in vitro and in vivo in LNCaP-tumor bearing mice and healthy pigs [136]. [^68^Ga]Ga-Glu-urea-Lys-HBED-CC-IRDye800CW showed an increased tumor uptake in mice compared to [^68^Ga]Ga-PSMA-11 (13.66 ± 3.73% ID/g vs. 4.89 ± 1.34% ID/g) [136,137]. Non-radiolabeled Glu-urea-Lys-HBED-CC-IRDye800CW has been investigated in healthy pigs using a DaVinci robotic system. A PSMA-specific fluorescence signal has been shown in vivo and ex vivo for both mice and pigs. More recently, the linker design of Glu-urea-Lys-HBED-CC-IRDye800CW was modified by insertion of histidine- and glutamic acid spacers ((HE)_3_) near the PSMA binding motif to further reduce background uptake in non-target tissue and to accelerate excretion [138,139]. Glu-urea-Lys-(HE)_3_-HBED-CC-IRDye800CW (PSMA-914) was used in a proof-of-concept study, with a first, pre-operative PET/CT imaging, 1-day prior surgery in a patient with high-risk prostate carcinoma and a second PET/CT imaging, after the surgical removal of the primary tumor [140]. ^68^Ga-radiolabeled PSMA-914 and PSMA-914 alone were administered 1-h prior to PET imaging and DaVinci robot-assisted prostatectomy respectively.

Schottelius et al. conjugated the disulfonated analog of the far-red cyanine dye Cy5 to PSMA I&T to obtain the hybrid imaging agent PSMA I&F (DOTAGA-*D*-Lys(N_ε_-Sulfo-Cy5)-*D*-(3-iodo)Tyr-*D*-(3-iodo)Tyr-*D*-Lys(N_ε_-Sub-KuE) [141]. Tumor uptake in LNCaP xenograft-bearing mice was comparable between [^68^Ga]Ga-PSMA I&F and [^68^Ga]Ga-PSMA I&T (4.5 ± 1.8% ID/g vs. 4.9 ± 1.6% ID/g) while kidney uptake was almost doubled for [^68^Ga]Ga-PSMA I&F (105.8 ± 22.7% ID/g vs. 53.3 ± 9.0% ID/g).

One limitation/challenge regarding the utilization of fluorescent dyes is that the detection efficacy of tumor lesions is limited to superficial tissue [142,143]. Certain dyes can also be used for photodynamic therapy [144]. In this approach, a dye can also simultaneously act as a photosensitizer which is selectively activated with long wavelength light, subsequently causing reactive oxygen species (ROS) formation leading to the induction of immediate cell death [108]. Derks et al. synthesized a variety of Glu-urea-Lys-based PSMA-targeting ligands conjugated to IRDye700DX [145]. The most promising candidate PSMA-N064 consists of a DOTAGA chelator, additional glutamic acid residues in the linker structure, a Glu-urea-Lys binding motif, and a fluorophore. After radiolabeling of PSMA-N064 with ^111^In, an increased tumor uptake of up to 2 h post injection (13.1 ± 2.3% ID/g) was determined in mice bearing LS1754T colon carcinoma cells transfected with human PSMA (LS1754T-PSMA). The tumors could be visualized using SPECT/CT and near infrared fluorescence scanners. Interestingly, the incorporation of the fluorophore leads to a higher internalization in tumor cells than the corresponding ligand without the dye (15.1 ± 0.8% ID/g vs. 6.7 ± 1.1% ID/g). Accordingly, the fluorophore parts may have a positive impact on tumor uptake [136,145]. Samples obtained from biopsies of human normal tissue could be distinguished from tumor tissue after incubation with PSMA-N064 using fluorescence imaging. In a proof-of-concept study for targeted photodynamic therapy, the incubation of LS1754T-PSMA cells with PSMA-N064 and exposure to NIR light resulted in reduced cell viability (34 ± 3.2%).

## 4. Multitargeted PSMA Inhibitors

### 4.1. PSMA + Albumin

PSMA inhibitors used for endoradiotherapy, such as PSMA-617 and PSMA I&T are rapidly excreted via the kidneys, increasing the radiation exposure to these organs [146]. Introducing albumin-binding groups into the chemical structure of small molecules can increase their blood circulation time, which can potentially lead to enhanced tumor uptake, while the reduction of injected activity results in lower non-target tissue doses [147,148].

The 4-(*p*-iodophenyl)acetic acid moiety was employed as an albumin-binding group by Kelly et al. in radiotracers bearing a DOTA chelator and a urea-based PSMA binding-motif radiolabeled with ^131^I (RPS-025) and ^177^Lu (RPS-063 & RPS-067). This modification lead to a 4-fold higher tumor uptake compared to [^177^Lu]Lu-PSMA-617, however, kidney-uptake was still significantly high [149,150]. It was observed that the use of polyethylene glycol (PEG) linkers of varying lengths had a dramatic impact on PSMA binding affinity as well as blood clearance [150]. Replacement of the albumin-binding group 4-(*p*-iodophenyl)acetic acid moiety with 4-(*p*-iodophenyl)butanoic acid (RPS-072) further reduced radiation exposure to the kidney and blood clearance [151].

Benešová et al. used the 4-(*p*-iodophenyl)butanoic acid moiety as an albumin-binding group in a similar way and extended the linker entity with aspartate residues to counterbalance the lipophilicity of the albumin-binding group [152]. Three radioligands consisting of a DOTA chelator, the aforementioned linker, the albumin-binding group and a urea-based PSMA binding motif (PSMA-ALB-02, PSMA-ALB-05 and PSMA-ALB-97) were radiolabeled with ^177^Lu and evaluated against [^177^Lu]Lu-PSMA-617 in PC-3 PIP tumor bearing mice with all three ligands showing a high tumor-to-blood ratio but still high kidney uptake. These results were further optimized by the replacement of the 4-(*p*-iodophenyl)butanoic acid moiety with (*p*-tolyl)butanoic acid [153], as the latter demonstrated a reduced albumin-binding affinity [147]. The most promising candidate PSMA-ALB-56 was compared against PSMA-617 in a therapy study of PC-3 PIP tumor bearing mice. [^177^Lu]Lu-PSMA-ALB-56 showed a better median survival time of the treated mice than [^177^Lu]Lu-PSMA-617 when the same activity was applied (5 MBq/mouse), with 4/6 of the [^177^Lu]Lu-PSMA-ALB-56-treated mice surviving the study. The use of 2-PMPA improved the tumor-to-kidney ratio by reducing kidney uptake of [^177^Lu]Lu-PSMA-ALB-56 [154]. In a recently published clinical study, ten patients with metastatic castration resistant prostate cancer (mCRPC) received [^177^Lu]Lu-PSMA-ALB-56 endoradiotherapy with a higher absorbed dose in tumor lesions and similar salivary gland-uptake compared to PSMA-617 and PSMA I&T [155]. Nevertheless, kidney and red marrow uptake in these patients remains high, this demands further preclinical optimization of albumin-binding PSMA radioligands (e.g., upon utilization of ibuprofen as an albumin-binding entity) [156,157,158].

### 4.2. PSMA + GRPr

Apart from PSMA, gastrin-releasing peptide receptors (GRPr) are frequently highly expressed in PCa tumors. The bombesin peptide (BBN = Pyr^1^-Gln^2^-Arg^3^-Leu^4^-Gly^5^-Asn^6^-Gln^7^-Trp^8^-Ala^9^-Val^10^-Gly^11^-His^12^-Leu^13^-Met^14^-NH_2_) is considered a potent agonist for GRPr, while over the years several BBN analogs (either agonists or antagonists of GRPr) have been radiolabeled and evaluated for potential application in Nuclear Medicine [159,160]. The expression of both receptors in tumors is heterogeneous [161], a fact that could potentially result in reduced image quality or false-negative results. A recent preclinical study, which evaluated retrospectively frozen prostatectomy samples (*n* = 20) with tissular microimaging using [^111^In]In-PSMA-617 and the GRPr antagonist [^111^In]In-RM2 ([^111^In]In-DOTA-4-amino-1-carboxymethyl-piperidine-*D*-Phe^6^-Gln^7^-Trp^8^-Ala^9^-Val^10^-Gly^11^-His^12^-Sta^13^-Leu^14^-NH_2_), showed that for [^111^In]In-PSMA-617 the binding was high and independent of the metastatic risk (*p* = 0.665), Gleason score (*p* = 0.555), or PSA value (*p* = 0.404), while [^111^In]In-RM2 exhibited a significantly higher binding in the low metastatic risk group (*p* = 0.046), in the low PSA value group (*p* = 0.001), and in samples with Gleason 6 score (*p* = 0.006) [162]. A limited number of patients have been screened with a PSMA-targeting imaging agent ([^68^Ga]Ga-PSMA-11 or [^18^F]DCFPyL) and the GRPr antagonist [^68^Ga]Ga-RM2 [163,164,165,166,167] resulting rather in a complementing than in a competing approach of PSMA vs. GRPr imaging [165,168].

However, in order to overcome this limitation, and increase specificity and sensitivity of the imaging method, the heterodimers approach has been described for a combination of various targets [169,170]. Specifically, heterodimers (Hd) targeting PSMA/GRPr have been recently reviewed by Liolios et al. [171]. The first reported heterodimer targeting both PSMA and GRPr consisted of the acyclic chelator HBED-CC, which was linked to either side via its two carboxylic groups not participating at the complexation of the radiometal with the Lys-CO-Glu-OH PSMA binding motif and the GRPr agonist H_2_N-PEG_2_-[*D*-Tyr^6^,*beta*-Ala^11^,Thi^13^,Nle^14^)BN(6–14) [172]. The BBN analog, in this case, was structurally relevant to [^68^Ga]GaBZH_3_ (DOTA-PEG_2_-[*D*-tyr^6^, *beta*-Ala^11^, Thi^13^, Nle^14^] BN(6–14)), which contained a modified BBN sequence, resistant to peptidases, and which has been clinically tested [173,174]. The heterodimer Glu-urea-Lys(Ahx)-HBED-CC-BZH3 (Hd-**1**) showed affinity values comparable to the monomers (PSMA IC_50_ = 25 ± 5.4 nM, GRPr IC_50_ = 9.0 ± 1.8 nM) during in vitro testing in PC-3 (GRPr-positive, androgen independent) and LNCaP (PSMA-positive, androgen depended) cells. Specific cell-binding (either surface bound or internalized) for the ^68^Ga labeled heterodimer was similar to the control ^68^Ga-monomers, while tumor uptake in LNCaP and AR42J (GRPr positive) xenografts were 5.44 ± 1.54% ID/g and 3.34 ± 1.05% ID/g, respectively at 1 h p.i. Tumors were clearly visible during micro-PET imaging (1 h p.i.), however, a high kidney uptake was noted (110.46 ± 8.80% ID/g, 1 h, p.i.), which was minimized during PSMA block (16.04 ± 3.94% ID/g), but remained unaffected during GRPr block (109.52 ± 34.52% ID/g) clearly indicating the role of PSMA receptors regarding kidney accumulation. This heterodimer was improved to reduce kidney uptake by the addition of so-called (HE)_n_ (*n* = 1–3) amino acid spacers between the HBED-CC chelator and the PSMA pharmacophore [138]. The new heterodimers (HE_n_, where *n* = 0, Hd-**2**, *n* = 1, Hd-**3**, *n* = 2, Hd-**4**, *n* = 3, Hd-**5**), when tested in vitro they showed high affinity in all cell lines tested: PC-3 (IC_50_ = 4.40 to 7.72 nM), AR42J (IC_50_ = 2.58 to 5.06 nM) and LNCaP (IC_50_ = 17.4 to 42.4 nM), which were comparable to the respective monomers for each case (PC-3, 3.65 nM, AR42J, 1.29 nM, LNCaP 7.5 nM). During the in vivo experiments in mice with PC-3 ad LNCaP xenografts the HE_2_ spacer in addition to the reduction of kidney uptake, also managed to increase tumor uptake for both xenografts (Table 3).

Bandari et al. synthesized and radiolabeled with the PET radionuclide ^64^Cu, the heterodimer: DUPA-6-Ahx-(^64^Cu-NODAGA)-5-Ava-BBN(7–14)NH_2_ [175]. The heterodimer (Hd-**6**) was evaluated in vitro in PC-3 and LNCaP cells, where it showed high affinity (PC-3: IC_50_ = 11.1 ± 0.46 nM; LNCaP: IC_50_ = 1.16 ± 1.35 nM), while during in vivo MicroPET scintigraphy the xenografted PC-3 and LNCaP tumors were clearly visible at 18 h p.i. Bandari et al. also synthesized the heterodimer [DUPA-6-Ahx-Lys(DOTA)-6-Ahx-RM2] using the antagonist BN peptide (BBN ANT) sequence of RM2 (BBN ANT = D-Phe^6^-Gln^7^-Trp^8^-Ala^9^-Val^10^-Gly^11^-His^12^-Sta^13^-Leu^14^-NH_2_) (Hd-**7**), which was labeled with ^68^Ga, ^111^In, or ^177^Lu for theranostic applications [176]. In a later study and based on this structure, Bandari et al. synthesized and evaluated several heterodimeric ligands testing different spacer groups in addition to 6-Ahx connecting the BBN peptide with the chelator DO3A (DUPA-6-Ahx-[DO3A]-X-BBN ANT, where DO3A = 1,4,7,10-tetraazacyclododecane-1,4,7-triacetic acid and X = 5-aminovaleric acid, 5-Ava; 6- aminohexanoic acid, 6-Ahx; 8-aminooctanoic acid, 8-Aoc; and para-aminobenzoic acid, AMBA). In vitro testing revealed low IC_50_ values for PC-3 (4–7 nM), and LNCaP cells (10–16 nM), both before and after metalation with ^nat^In and ^nat^Lu [177]. Among the different candidates, the heterodimer DUPA-6-Ahx-[DO3A]-8-Aoc -BBN ANT (Hd-**8**) was selected as the optimal candidate for further in vivo testing with PC-3 and LNCaP xenografts. The selection criterion was its higher accumulation in the pancreas (5.45 ± 0.47% ID/g, 1 h p.i.) during biodistribution studies in normal mice, which clearly indicated GRPr targeting since the normal pancreas is known to overexpress GRPr receptors. The selected heterodimer after radiolabeling with ^111^In and ^177^Lu presented high stability, both in PBS and in human serum after 4 h of incubation (at RT), while minor radiolytic degradation was observed at the 24 h. In vivo biodistribution experiments in mice carrying PC-3 or PC-3 PIP xenografts showed rapid elimination (85% of the injected dose, 4 h p.i.) of each tracer (^111^In or ^177^Lu) from the bloodstream with excretion profiles being primarily via the renal-urinary pathway. Kidney uptake was also high ranging from 8.90 ± 1.40% ID/g to 32.2 ± 28.8% ID/g (until 4 h p.i.), which was also expected considering PSMA expression in this organ. Tracer uptake in xenografted tumors ranged from 4.74 ± 0.90 to 7.51 ± 2.61% ID/g, with the highest values observed at 1 h p.i. and gradually decaying thereafter. Higher tumor values were observed for the ^177^Lu labeled compound in both xenografts, which according to the authors was due to the presence of more nonradioactive [DUPA-6-Ahx-([^nat^Lu]-Lu-DO3A)-8-Aoc-BBN ANT] in comparison to the ^111^In labeled analog [177]. Possibly, the nonradioactive ligand blocked the clearance route of the radioactive tracer, resulting in higher kidney radioactivity and more importantly in higher circulating amounts of [DUPA-6-Ahx-([^177^Lu]-Lu-DO3A)-8-Aoc-BBN ANT], which eventually was accumulated in the tumors. This hypothesis in combination with the increased radioactivity amounts observed in normal organs during the blocking experiments further noted the importance of high specific activity for such radiopharmaceutical applications and further enhancement of heterodimeric ligands, i.e., with albumin binders.

Mendoza-Figueroa et al. designed a heterodimeric ^68^Ga-labeled radiotracer ([^68^Ga]Ga-iPSMA-BN) (Hd-**9**) consisting of an iPSMA (Nal-Lys-CO-Glu-OH) and a Lys^3^-Bombesin part linked to a DOTA chelator. When [^68^Ga]Ga-iPSMA-BN was evaluated against the monomers [^68^Ga]Ga-iPSMA and [^68^Ga]Ga-BN in vitro and in vivo in LNCaP cells and PC-3 cells, it showed superiority against each monomer [^68^Ga]Ga-iPSMA and [^68^Ga]Ga-BN, in both cell lines and animal models (Table 3) [178]. The same group has published a study where they evaluated this heterodimer labeled with ^177^Lu [179]. The heterodimer showed a significant decrease in LNCaP (10.15%) and PC-3 (40.10% at 48 h) viability in vitro, and a high LNCaP and PC-3 tumor uptake in ex vivo biodistributions (5.21 and 3.21% ID/g at 96 h, respectively), and Micro-SPECT/CT imaging studies (SUV_max_ of 1.93 ± 0.30 and 1.76 ± 0.10 in LNCaP and PC-3, respectively), possibly influenced by the heterobivalent effect. More recently, biokinetics and dosimetry data have been obtained for [^68^Ga]Ga-iPSMA-BN in a study of four healthy patients showing specific uptake in the pancreas (GRPr expressing) and salivary glands (PSMA expressing) [180].

Mitran et al. synthesized the heterodimer NOTA-DUPA-RM26 (BQ7800) consisting of (Hd-**10**) (DUPA = 2-[3-(1,3-dicarboxypropyl)-ureido]pentanedioic acid) consisting of a Glu-CO-Glu PSMA-binding motif and the GRPr antagonist RM26 (D-Phe-Gln-Trp-Ala-Val-Gly-His-Sta-Leu-NH2). Both binding motifs were coupled via glutamic acid and contain NOTA as a chelator for radiolabeling with either ^68^Ga or ^111^In [181]. In vitro preclinical evaluation resulted in high affinity for GRPr (IC_50_ = 4.0 ± 1.0 nM) and rather low for PSMA (IC_50_ = 824 ± 230 nM). However, at 1 h p.i., tumor uptake in PC-3 PIP xenografts for [^111^In]In-BQ7800 (12 ± 2% ID/g) and [^68^Ga]Ga-BQ7800 (8 ± 2% ID/g) was higher than the one in normal organs and was decreased thereafter. However, kidney uptake was similar to the tumor uptake, while increased uptake was observed in organs expressing GRPr receptors. Attempts to improve the PSMA-specific tumor uptake were made by incorporating phenylalanine into the linker of the PSMA-binding motif and shortening of the PEG linker (BQ7812). The tumor uptake of [^111^In]In-BQ7812 (Hd-**11**) was doubled compared to [^111^In]In-BQ7800, but the modifications also led to a three-fold increase of uptake in the kidneys [182].

Finally, three additional bispecific heterodimers based on RM2 peptide sequence (*D*-Phe^6^-Gln^7^-Trp^8^-Ala^9^-Val^10^-Gly^11^-His^12^-Sta^13^-Leu^14^-NH_2_) and PSMA-617 are reported [183]. The two pharmacophores were linked via the spacer: X-triazolyl-Tyr-PEG_2_, where X = 0 (Hd-**12**), PEG_2_ (Hd-**13**), (CH_2_)_8_ (Hd-**14**). The resulting heterodimers were radio-iodinated and evaluated in vitro for binding specificity, cellular retention, and affinity. In vivo specificity and tumor uptake for all heterodimers was studied in PC-3 and LNCaP xenografts (Table 3), while [^125^I]I-Hd-**13** was also evaluated in PC-3 PIP xenografts where it showed high tumor accumulation (30– 35% ID/g at 3 h p.i.). However, this was followed by high kidney radioactivity values like the first heterodimer: [^68^Ga]Ga-Hd-**1**.

**Table 3:** Heterodimers targeting PSMA/GRPr, tumor uptake (1 h p.i.) expressed as (% ID/g) in PC-3, AR42J, LNCaP or PC-3 PIP xenografts (Mean value ± Std).

Heterodimer	Tumor Uptake (1 h p.i.; ^&^24 h p.i., ^#^ 96 h p.i.) (% ID/g ± SD)				Ref.
	PC-3	AR42J	LNCaP	PC-3 PIP	
[^68^Ga]Ga-Hd-1	-	3.34 ± 1.04	5.44 ± 1.54	-	[172]
[^68^Ga]Ga-Hd-2	0.84 ± 0.18	-	2.38 ± 0.05	-	[138]
[^68^Ga]Ga-Hd-3	2.67 ± 1.25	-	2.41 ± 1.24	-	
[^68^Ga]Ga-Hd-4	2.23 ± 0.27	-	10.66 ± 4.19	-	
[^68^Ga]Ga-Hd-5	1.66 ± 0.41	-	3.22 ± 0.22	-	
[^64^Cu]Cu-Hd-6	-	-	-	-	[175]
Hd-7	-	-	-	-	[176]
([^111^In]In-Hd-8	4.74 ± 0.90	-	-	5.38 ± 1.07	[177]
[^177^Lu]Lu-Hd-8	7.51 ± 2.61	-	-	7.37 ± 2.89	[177]
[^68^Ga]Ga-Hd-9	7.39 ± 0.65	-	13.67 ± 3.88	-	[178]
[^177^Lu]Lu-Hd-9	1.76 ± 0.10 ^&^ ~ 3.21 ^#^	-	1.93 ± 0.3 ^&^ ~ 5.21 ^#^	-	[179]
[^111^In]In-Hd-10	-	-	-	12.0 ± 2.0	[181]
[^68^Ga]Ga-Hd-10	-	-	-	8.0 ± 2.0	[181]
[^111^In]In-Hd-11	-	-	-	16.10 ± 2.96	[182]
[^125^I]I-Hd-12	3.0 ± 0.3		11.0 ± 3.0		[183]
[^125^I]I-Hd-13	4.3 ± 0.6		10.0 ± 3.0		
[^125^I]I-Hd-14	2.2 ± 0.6		3.0 ± 2.0	~ 21.0	

### 4.3. PSMA + Integrin α_v_β_3_

Two heterobivalent imaging agents targeting PSMA and integrin-α_v_β_3_ surface markers, both overexpressed in certain tumor epithelium and/or neovasculature were synthesized and evaluated by Shallal et al. [184]. Integrins α_v_β_3_ are considered an ideal target for the development of radioligands, since they are overexpressed on tumor vasculature due to angiogenesis, and on the cell membrane in various tumors, i.e., ovarian cancer, neuroblastoma, breast cancer and melanoma [185]. The tripeptide RGD (arginine-glycine-aspartic) amino acid sequence and its cyclic RGD (cRGD) analogs specifically bind to the integrin α_v_β_3_ receptor and thus have provided the platform for the development of various radioligands [185]. The heterodimers consisted of Lys-CO-Glu-OH PSMA binding motif connected to the Lys of a cyclic cRGDfK peptide (cyclo-(Arg-Gly-Asp-*D*-Phe-Lys)) and coupled either to the DOTA chelator for radiolabeling (Hd-**15**) or to the dye IRDye800CW (Hd-**16**). In vitro testing in PC-3 PIP and U87-MG cells and in isolated proteins showed low affinity with high IC_50_ values for the both PSMA and α_v_β_3_ receptors, i.e., Hd-**15:** PC-3 PIP, IC_50_ = 479 nM, U87-MG, IC_50_ = 1536 nM, Hd-**16:** integrin α_v_β_3_ IC_50_ = 90 nM. In vivo, optical imaging and ex vivo biodistribution studies for Hd-**16** showed specific tumor uptake for PC-3 PIP tumors expressing PSMA, and U87-MG tumors expressing integrin α_v_β_3_. Tumor uptake was dose-dependent, and even at the lowest dose (0.5 nmol) the tumors were clearly visible. However, this study did not include a metalated or a radiolabeled version of Hd-**15** [184].

## 5. Future Methods and Concepts for PSMA Inhibitor Radiolabeling

The fluoroglycosylation method by Maschauer et al. [186] has been applied to obtain 2-[^18^F]FGlc-PSMA and 6-[^18^F]FGlc-PSMA with the latter example highlighting a 10-fold decrease in kidney uptake in PC-3 PIP tumor bearing mice compared to 2-[^18^F]FGlc-PSMA and [^68^Ga]Ga-PSMA-11 [187].

Greifenstein et al. used a squaric acid (SA) moiety to link the Glu-urea-Lys PSMA binding motif with the chelator [188]. Out of three SA-PSMA inhibitors with different chelators, DOTAGA.SA.PSMA emerged as a lead structure to be compared against ^68^Ga-radiolabeled PSMA-617 and PSMA-11. [^68^Ga]Ga-DOTAGA.SA.PSMA showed tumor uptake in LNCaP tumor bearing mice comparable with [^68^Ga]Ga-PSMA-617 and lower than [^68^Ga]Ga-PSMA-11 (5.6 ± 0.3% ID/g vs. 6.5 ± 1.0% ID/g vs. 12.8 ± 1.5% ID/g), but significantly lower uptake in kidneys (3.2 ± 0.5% ID/g vs. 16.2 ± 5.7% ID/g vs. 210.8 ± 8.1% ID/g) and in other organs (60 min p.i.) showing thus a potential as therapeutic agent [189].

The isotopic exchange chemistry for the fluorine isotopes ^19^F/^18^F has been described by Schirrmacher et al. using silicon-fluoride-acceptor (SiFA) building blocks [190]. This concept has been adopted for PSMA inhibitors, which also introduce a chelator into the molecule in close proximity to the SiFA motif in order to increase hydrophilicity [191]. This permits the radiolabeling of the molecule with either ^18^F at the SiFA building block or with a radiometal of choice (e.g., ^68^Ga, ^177^Lu, ^225^Ac) at the chelator while the moiety not used for radiolabeling is occupied with the non-radioactive ^19^F or metal, which has been called by the authors radiohybrid PSMA (rhPSMA) inhibitors. Wurzer et al. developed a series of these rhPSMA inhibitors, with rh-PSMA-7 being the most promising candidate being radiolabeled with ^18^F, bearing ^nat^Ga within the chelator and being clinically evaluated in a number of patients with primary and biochemical recurrence of PCa [191,192,193,194]. Further improvement of rh-PSMA-7 has been achieved by investigation of its four stereoisomers and their influence on pharmacokinetics [83]. The resulting lead candidate [^18^F]Ga-rh-PSMA-7.3 showed the highest tumor accumulation and low uptake in non-target tissues. This was confirmed in healthy volunteers determining biodistribution and radiation dosimetry data (NCT03995888) and in a larger cohort of patients [84,195,196,197]. The molar activity of [^18^F]Ga-rh-PSMA-7.3 had a minor influence on the biodistribution [198,199]. Three clinical trials using [^18^F]Ga-rh-PSMA-7.3 are either ongoing or will commence in the near future (NCT04978675/NCT04186819/NCT04186845). The therapeutic counterpart [^177^Lu]Lu-rhPSMA-7.3 showed a higher anti-tumor effect and longer median survival in C4-2 xenograft-bearings SCIDS than [^177^Lu]Lu-PSMA I&T [85] followed by a pre-therapeutic dosimetry study in six mCRPC patients confirming the preclinical results [86].

Another approach of isotopic exchange has been reported for eight PSMA inhibitors by substituting ^19^F with ^18^F at a trifluoroborate group [200]. This concept has been extended to the radiohybrid ligand DOTA-AMBF_3_-PSMA [201]. The PSMA-617 pharmacophore was used as a starting point, then a lysine-trifluoroborate moiety was introduced and linked to a DOTA-chelator allowing radiolabeling with either ^18^F or a radiometal. DOTA-AMBF_3_-PSMA radiolabeled with ^18^F/free, ^18^F/^nat^Cu, ^nat^F/^64^Cu and ^nat^F/^177^Lu was preclinically evaluated in LNCaP xenografts with tumor uptake > 10% ID/g for all radiotracers. [^64^Cu]Cu-DOTA-AMBF_3_-PSMA showed increased liver uptake, most likely due to transchelation of ^64^Cu from the DOTA-chelator which has also been observed for [^64^Cu]Cu-DOTA and [^64^Cu]Cu-PSMA-617 [202,203]. An increased liver uptake was seen in patients scanned with [^64^Cu]Cu-PSMA-617 2 h and 22 h post injection as well as in a biodistribution and radiation dosimetry study using [^64^Cu]CuCl_2_ [204,205].

## 6. Potential Radionuclides for the Future Use of PSMA Inhibitors

While radionuclides like ^68^Ga, ^18^F, or ^177^Lu have become a sort of routine in the use of radiolabeled PSMA inhibitors, other radionuclides are gaining attention due to their specific properties [206,207,208]. For example, ^225^Ac can play a pivotal role in targeted alpha therapy (TAT) of prostate cancer [48,51,78,209,210,211,212,213,214,215], but additional preclinical studies and prospective clinical trials need to be performed to secure its safety and efficacy [216,217,218,219,220,221]. The importance of ^225^Ac-labeled radiopharmaceuticals is affirmed by efforts to establish GMP-compliant procedures for production and quality control [222,223,224,225,226]. Regulatory aspects on the supply of radionuclides for routine clinical application represent a hurdle that needs to be considered for the translation of new radiopharmaceuticals [227,228].

### 6.1. ^211^Astatine

^211^At is a radiohalogen with a half-life of 7.2 h suitable for TAT using small molecules. Several PSMA radioligands have been radiolabeled with ^211^At and indicated improved survival rates in PC-3 PIP PSMA-positive tumor bearing mice [229,230]. Although the inclusion of albumin binding moieties increases blood retention and reduces kidney uptake [149], dehalogenation and nephrotoxicity have been the limiting factors for clinical translation. The latest development ^211^At-3-Lu [230] showed improved toxicity data for kidney uptake and reduced off-target uptake in organs like the stomach and salivary glands. The infrastructure for the production of ^211^At-labeled radiopharmaceuticals is complex though, efforts are made to make them more widely available [231,232], such as the NOAR COST action, which has the goal to create a European Network for Optimized Astatine labeled Radiopharmaceuticals.

### 6.2. ^64/67^Copper

The Donnelly group investigated the bivalent PSMA inhibitor Sar-bisPSMA with the matched pair ^64^Cu/^67^Cu against [^68^Ga]Ga-PSMA-11 and [^177^Lu]Lu-PSMA I&T respectively in a LNCaP xenograft model [233,234]. Sar-bisPSMA consists of a sarcophagine based chelator covalently linked to two lysine-ureido-glutamate functional groups, which can be radiolabeled with copper isotopes at room temperature. [^64^Cu]Cu-Sar-bisPSMA showed higher tumor uptake than [^68^Ga]Ga-PSMA-11 1 h after injection (9 ± 1% IA/g vs. 5 ± 1% IA/g). The dose-dependent tumor growth inhibition of [^67^Cu]Cu-Sar-bisPSMA was comparable to [^177^Lu]Lu-PSMA I&T (5 MBq: 58 vs. 65%, 30 MBq: 109 vs. 107% against vehicle control). The longer half-life of [^64^Cu]Cu-Sar-bisPSMA allows prospective dosimetry for the use of [^67^Cu]Cu-Sar-bisPSMA. Two clinical trials using [^64^Cu]Cu-Sar-bisPSMA and [^67^Cu]Cu-Sar-bisPSMA have been started in 2021 (NCT04839367/NCT04868604).

Kelly et al. similarly used a sarcophagine chelator (MeCOSar) conjugated to N^ε^-(2-(4-iodophenyl)acetyl)lysine as albumin binding group and a PSMA binding motif (RPS-085) [235]. Uptake of [^64^Cu]Cu-RPS-085 in LNCaP tumor bearing mice was constant for the tumor (4 h p.i.: 12.9 ± 1.4% ID/g, 24h p.i.: 8.3 ± 0.8% ID/g, 48 h p.i.: 9.8 ± 1.3% ID/g), while the uptake in the kidneys decreased over time leading to an increased tumor-to-kidney ratio of 6.1 ± 0.8 48 h p.i. The biodistribution was similar when RPS-085 was radiolabeled with ^67^Cu, with a tumor-to-kidney ratio of 3.0 ± 0.5, 96 h p.i.

### 6.3. ^203/212^Lead

^203^Pb and ^212^Pb form an ideal theranostic pair for SPECT imaging and TAT with half-lives of 51.9 h and 10.6 h respectively. The production methods for ^203/212^Pb have been improved making them available for potential clinical application [236]. Dos Santos et al. developed four PSMA inhibitors (CA008, CA009, CA011 and CA012) with *p*-SCN-Bn-TCMC and DO3AM chelators bearing the PSMA-617 binding motif and linker structure [237]. The best ligand PSMA-CA012 radiolabeled with ^203^Pb showed similar tumor uptake like [^68^Ga]Ga-PSMA-617 in PSMA-positive C4-2 tumor-bearing mice (8.4 ± 3.7% ID/g vs. 8.5 ± 4.1% ID/g). Two patients have been studied first-in-human with [^203^Pb]Pb-PSMA-CA012 and safety dosimetry estimates for [^203^Pb]Pb-PSMA-CA012 and [^212^Pb]Pb-PSMA-CA012 were determined. Stenberg et al. reported the synthesis of NG-001, which has the same chemical structure as CA009 [238]. [^212^Pb]Pb-NG001 was prepared in situ from a ^224^Ra-solution in equilibrium with progeny [239]. [^212^Pb]Pb-NG001 was evaluated against [^212^Pb]Pb-PSMA-617 in mice bearing C4-2 tumors. While the tumor uptake was comparable 2 h p.i. (17.61 ± 6.76% ID/g vs. 17.93 ± 2.90% ID/g), kidney uptake was lower for [^212^Pb]Pb-NG001 (21.07 ± 10.33% ID/g vs. 52.82 ± 26.62% ID/g) [238,239]. Dose-dependent treatment efficiency of [^212^Pb]Pb-NG001 was confirmed in multicellular C4-2 spheroids and the corresponding mouse model with no long-term toxicity effects [240]. Apart from DOTA and TCMC, pyridine-based cyclen analogs (DOTA-1Py, DOTA-2Py and DOTA-3Py) [236] may be a better chelator alternative for ^212^Pb and its daughter isotopes for avoiding recoil effects [241].

### 6.4. ^149/152/155/161^Terbium

Terbium, also called the “Swiss Army Knife” of Nuclear Medicine with its quadruplet of radioisotopes [242] with suitable properties for imaging with PET (^152^Tb/^149^Tb) and SPECT (^155^Tb) and endoradiotherapy with alpha (^149^Tb) and beta^−^/Auger electrons (^161^Tb), has been investigated by the group at Paul Scherrer Institute (PSI). Terbium radioisotopes have been used in combination with PSMA-617 in PSMA-positive PC-3 PIP tumor cells. [^152^Tb]Tb-PSMA-617 has been evaluated in vitro and in vivo and successfully applied first-in-human in a patient with metastatic castration-resistant prostate cancer [76]. Suppression of tumor growth has been demonstrated using [^149^Tb]Tb-PSMA-617 in TAT in mice bearing PC-3 PIP tumor cells [243]. In a preclinical study, [^161^Tb]Tb-PSMA-617 was compared against [^177^Lu]Lu-PSMA-617 in a PC-3 PIP xenograft model showing the superiority of ^161^Tb vs. ^177^Lu [244,245] opening the way for clinical translation. The additional therapeutic effect of ^161^Tb due to Auger electrons was confirmed by a computational approach using a microdosimetry model [246]. However, despite these very promising results, the production of terbium radioisotopes for clinical application remains challenging [247,248].

### 6.5. ^89^Zirconium

Vázquez et al., modified PSMA-617 by replacing DOTA with DFO as chelator making it suitable for radiolabeling with ^89^Zr [249]. ^89^Zr is a positron-emitter with a physical half-life of 3.27 days. The aim of this study was to detect low PSMA-expressing lesions which are negative on PSMA PET scans using short-lived radiotracers, such as [^68^Ga]Ga-PSMA-11 and [^18^F]JK-PSMA-7 by increasing the time for internalization of the radioligand. In a LNCaP xenograft model, 24 h after intravenous injection [^89^Zr]Zr-PSMA-DFO showed a higher tumor-to-background ratio than [^68^Ga]Ga-PSMA-11 and [^18^F]JK-PSMA-7 after 2 and 4 h. In a small number of patients (8/14) additional lesions were identified with [^89^Zr]Zr-PSMA-DFO after initial scans were negative with [^68^Ga]Ga-PSMA-11 or [^18^F]JK-PSMA-7 [250]. An additional [^89^Zr]Zr-PSMA-DFO PET scan at later time points might be beneficial for patients with biochemical recurrence and weak PSMA expression in tumor lesions.

Noor et al. [251] conjugated the Lys-ureido-Glu binding motif to the desferrioxamine B squareamide ester derivative H_3_DFOSq, leading to two monovalent and two bivalent DFO-Sq-lysine-ureido-glutamate derivatives with different linker structures to be radiolabeled with ^89^Zr and ^68^Ga. The bivalent ligands performed superior in LNCaP tumor models compared to the monovalent ligands. The bivalent ligand bearing an aromatic hydrophobic linker (H_3_DFO-tren-bis(glut-PAMBA-Lys-ureido-Glu)) showed the highest tumor uptake when radiolabeled with ^89^Zr and ^68^Ga compared against [^68^Ga]Ga-PSMA-11 [252] 1 h after injection (9.33 ± 0.33% IA/g vs. 10.8 ± 1.3% IA/g vs. 4.89 ± 1.34% IA/g).

## 7. Conclusions

The feasibility of theranostics with PSMA-targeting radiopharmaceuticals has been successfully demonstrated in the last decade, upon their radiolabeling using pairs of radionuclides with suitable properties for imaging with PET or SPECT and endoradiotherapy with alpha and beta^−^/Auger electrons. Radioguided and fluorescence-guided surgery should be an additional treatment option for PCa patients, in this direction PSMA ligands for radio-/fluorescence-guided surgery and/or targeted photodynamic therapy are being investigated. In order to further improve the efficiency and efficacy of the existing PSMA radioligands for PCa diagnosis and radionuclide therapy several structural modifications have been suggested for reducing their uptake in off-target tissues or utilizing multitargeting (i.e., albumin, GRPr and integrin α_v_β_3_). Addressing additional targets like GRPr with one radiotracer could reduce the number of false-negative findings. New radionuclides in combination with PSMA-binding structures are also being explored, but care should be taken on their radiobiological effects, availability for mass production and cost.

## Figures and Tables

**Table 1:** PSMA-targeting radiopharmaceuticals marketed, under clinical investigation or in a clinical setting.

Radiopharmaceutical	Brand Name	Radionuclide	Reference	Number of Clinical Trials ^1^ (Completed/Ongoing)
DCFPyL	PYLARIFY	^18^F	[24,66] ^2^	14/51
PSMA-11	Illuccix ^3/^isoPROtrace-11	^68^Ga	[20,67] ^2,4^	24/89
	-	^18^F	[68,69]	2/-
PSMA-1007	-	^18^F	[35,70] ^4^	2/13
PSMA-617	-	^44^Sc	[71,72]	-/-
		^64^Cu	[73]	-/-
		^68^Ga	[49,74]	1/3
		^111^In	[75]	-/-
		^152^Tb	[76]	-/-
		^177^Lu	[49,77]	1/17
		^225^Ac	[78]	-/2
PSMA I&T	-	^68^Ga	[45]	-/-
		^111^In	[79,80]	-/1
		^177^Lu	[45]	-/5
		^225^Ac	[48]	-/-
PSMA I&S	-	^99m^Tc	[81]	-/3
MIP-1404	-	^99m^Tc	[39,82]	6/-
rhPSMA-7.3	-	^18^F	[83,84]	1/3
		^177^Lu	[85,86]	-/-
JK-PSMA-7	-	^18^F	[87,88]	-/-
THP-PSMA	GalliProst	^68^Ga	[89,90]	1/1
PSMA-R2	-	^68^Ga	-	1/1
		^177^Lu	-	0/1

^1^ According to clinicaltrials.gov accessed on 26 October 2021. ^2^ FDA-approved. ^3^ TGA-approved. ^4^ Ph. Eur. monograph applicable.

**Table 2:** Biodistribution data of [^177^Lu]Lu-PSMA-617 (5 pmoles) with co-injected non-radioactive PSMA-11 for tumor and selected non-target organs one hour after injection in athymic mice bearing PC-3 PIP tumors [106].

Added Amount of “Cold” PSMA-11 (Pmoles)	Tumor Uptake (% ID/g)	Kidney Uptake (% ID/g)	Salivary Gland Uptake (% ID/g)
0	21.71 ± 6.13	123.14 ± 52.52	0.48 ± 0.11
5	18.70 ± 2.03	132.31 ± 47.40	0.45 ± 0.15
100	26.44 ± 2.94	84.29 ± 78.25	0.38 ± 0.30
500	16.21 ± 3.50	2.12 ± 1.88	0.08 ± 0.03
1000	13.52 ± 3.68	1.16 ± 0.36	0.09 ± 0.07
2000	12.03 ± 1.96	0.64 ± 0.23	0.05 ± 0.02
